# Supplementary material for: A high-resolution mRNA expression time course of embryonic development in zebrafish
Source: eLife. 2017 Nov 16;6:e30860. doi: 10.7554/eLife.30860 (PMC5690287; doi:10.7554/eLife.30860)
Supplement: Supplementary file 6. [file elife-30860-supp6.zip › biolayout-clusters-files/Cluster183-genes.html]

Cluster183


# Cluster183: Genes

| Ensembl ID | Gene Name | Chr | Start | End | Biotype |
| --- | --- | --- | --- | --- | --- |
| ENSDARG00000074639 | ENSDARG00000074639 | 24 | 56416 | 69612 | protein\_coding |
| ENSDARG00000101462 | ENSDARG00000101462 | KN150699.1 | 8465 | 12431 | protein\_coding |
| ENSDARG00000098660 | TSTD2 | 14 | 51859817 | 51867252 | protein\_coding |
| ENSDARG00000020811 | efemp2b | 21 | 25551831 | 25568004 | protein\_coding |
| ENSDARG00000070794 | greb1 | 17 | 30352239 | 30400042 | protein\_coding |
| ENSDARG00000036359 | minal | 10 | 37571 | 42845 | protein\_coding |
| ENSDARG00000007582 | si:ch1073-155h21.1 | 20 | 109544 | 117377 | protein\_coding |
| ENSDARG00000089159 | si:dkeyp-89c11.2 | 4 | 25617903 | 25631536 | protein\_coding |
